# Larval trematode infections in *Lymnaea glabra* populations living in the Brenne Regional Natural Park, central France

**DOI:** 10.1051/parasite/2015038

**Published:** 2015-12-21

**Authors:** Daniel Rondelaud, Philippe Vignoles, Gilles Dreyfuss

**Affiliations:** 1 INSERM 1094, Faculties of Medicine and Pharmacy 87025 Limoges France

**Keywords:** Brenne Regional Natural Park, Cercaria, Digenean, *Lymnaea glabra*, Lymnaeidae, Snail

## Abstract

*Lymnaea glabra* is known to be a natural intermediate host of two flukes, *Calicophoron daubneyi* and *Fasciola hepatica*, in central France. But it can also sustain larval development of other digeneans. Adult snails were thus collected from 206 habitats in 2014 and 2015 to identify parasite species and determine the prevalence of each digenean infection in relation to the five types of snail habitats. Seven digenean species were noted in 321 infected snails (out of 17,647 *L. glabra*). Snails with *F. hepatica* or *C. daubneyi* were found in 14.5% and 12.6% of habitats, respectively. Percentages were lower for snails with *Opisthoglyphe ranae* (5.8%), *Haplometra cylindracea* (5.3%) and were less than 5% for those infected with *Echinostoma revolutum*, *Notocotylus sp.* or *Plagiorchis sp.* Prevalence noted for each parasite species varied with the type of habitat. The number of species in *L. glabra* was lower than that found in *G. truncatula* from the same region (7 instead of 10). The distribution and prevalence of each digenean species were thus dependent on the type and location of each snail habitat.

## Abstract (fr)

*Lymnaea glabra* est connu comme un hôte intermédiaire naturel de deux douves, *Calicophoron daubneyi* et *Fasciola hepatica*, dans le centre de la France. Mais cette limnée peut aussi soutenir le développement larvaire d’autres Digènes. Des *L. glabra* adultes ont été récoltés pour cela dans 206 habitats en 2014 et 2015 pour identifier les espèces de parasites et déterminer la prévalence de chaque Digène par rapport aux cinq types d’habitats à mollusques. Sept espèces de Digènes ont été trouvées chez 321 mollusques parasités (sur un total de 17 647 *L. glabra*). Les limnées avec *F. hepatica* ou *C. daubneyi* ont été récoltées, respectivement, dans 14.5 % et 12.6 % des habitats. Les pourcentages sont plus faibles pour les limnées avec *Opisthoglyphe ranae* (5.8 %), *Haplometra cylindracea* (5.3 %) et sont inférieurs à 5 % pour les mollusques infestés par *Echinostoma revolutum*, *Notocotylus sp.* ou *Plagiorchis sp.* La prévalence notée pour chaque espèce de parasite varie avec le type d’habitat. Le nombre d’espèces chez *L. glabra* est plus faible que celui trouvé chez les *Galba truncatula* de la même région (7 au lieu de 10). La distribution et la prévalence de chaque espèce de Digène sont donc dépendantes du type et de la localisation de chaque habitat à limnées.

Freshwater pulmonate gastropods play a significant role in the life cycles of many trematodes, as they ensure development of their larval forms. Examining these gastropods provides information on local parasitological fauna in a given region and on the sources of infection for definitive hosts [12, 31]. Among these gastropods, the family Lymnaeidae can sustain larval development of more than 70 trematode species [5, 24]. Contrary to other lymnaeids for which research on natural infection with trematode larvae has been conducted in Europe for over a hundred years [31], little information on parasitological fauna developing in the snail *Lymnaea glabra* O.F. Müller, 1774 [25] is available in the literature. This lymnaeid is known to be a natural intermediate host of two flukes: *Calicophoron daubneyi* Dinnik, 1962 [9] and *Fasciola hepatica* Linnaeus, 1758 [23] in central France [1–4]. But it can also sustain larval development of several other digeneans. Rondelaud [27] reported the presence of five digenean species in 13,039 *L. glabra* collected from several watercress beds located on the cristallophyllian and metamorphic soils of the Limousin region (central France). Overall prevalence was 4.3% for *F. hepatica*, 2.3% for *C. daubneyi*, 0.1% for *Haplometra cylindracea* Zeder, 1800 [32] and 0.09% for a total of two unidentified species. In contrast, in the swampy meadows of 18 farms located on the same types of soils, Abrous et al. [3, 4] reported the presence only of *F. hepatica* and/or *C. daubneyi* in *L. glabra*. In view of results reported by Rondelaud [27] and Abrous et al. [3, 4], it can be asked whether these findings would be the same in another French region: Did digenean prevalence and species composition change when the *L. glabra* samples were collected from a close sedimentary area with numerous meadows and ponds? Did prevalences, species composition and richness differ with the different types of snail habitats? To answer these two questions, adult snails were collected in 2014 and 2015 from five types of *L. glabra* habitats located in the Brenne Regional Natural Park, department of Indre (central France). After collection, snails were dissected to identify parasite species and determine prevalences of natural infections.

Snail investigations were carried out on six close municipalities: Chitray, Ciron, Migné, Nuret-le-Ferron, Rosnay and Ruffec. Five types of *L. glabra* habitats were identified in 2010–2011: (i) rainwater-draining furrows in meadows (37 habitats), (ii) temporary waterlogged road ditches (86), (iii) small permanent pools (< 50 m^2^) in meadows (28), (iv) banks of larger ponds near water inflows (31) and (v) brooks receiving water coming from upstream ponds (24) (Table 1). The soil of these habitats was composed of silt and sand, and was generally supported by sandstones and/or clay. In these 206 habitats, the total number of overwintering snails per habitat ranged from 84 to 202 adults at the beginning of field investigations (April, May or June 2014 according to the type of snail habitat). The temperate climate was frequently swept by humid winds which came from the West or the South-west [8]. The mean annual rainfall was about 800 mm and the mean annual temperature was 10.5°–11 °C with rather mild winters [10].

**Table 1.** Frequency of sites colonized by *Lymnaea glabra* and containing infected snails in relation to the type of habitat and digenean species. *n*, total number of habitats colonized by the lymnaeid.

Parasite species	Number of habitats containing infected snails (frequency in %)					
	Drainage furrows (*n*: 37)	Road ditches (*n*: 86)	Pools (*n*: 28)	Pond banks (*n*: 31)	Brooks (*n*: 24)	All sites (*n*: 206)
*Calicophoron daubneyi*	14 (37.8)	7 (8.1)	3 (10.7)	0	0	24 (11.6)
*Echinostoma revolutum*	0	0	0	2 (6.4)	0	2 (0.9)
*Fasciola hepatica*	17 (45.9)	11 (12.7)	1 (3.5)	1 (3.2)	0	30 (14.5)
*Haplometra cylindracea*	2 (5.4)	0	7 (25.0)	0	3 (12.5)	11 (5.3)
*Notocotylus* sp.	1 (2.7)	2 (0.2)	3 (10.7)	2 (6.4)	1 (4.1)	9 (4.3)
*Opisthoglyphe ranae*	0	0	5 (17.8)	7 (22.5)	0	12 (5.8)
*Plagiorchis* sp.	0	0	0	3 (9.6)	0	3 (1.4)
Immature infections						
with rediae	1 (2.7)	0	0	2 (6.4)	2 (8.2)	5 (2.4)
with sporocysts	3 (10.0)	1 (0.1)	0	3 (9.5)	1 (4.1)	8 (4.0)
Xiphidiocercariae (unidentified species)	0	0	7 (25.0)	2 (6.4)	1 (4.1)	10 (4.8)
Number of parasite species	4	3	5	5	2	7

Samples of 50 overwintering *L. glabra* (shell height, ≥ 8 mm) were randomly collected from each habitat in March (sites i–iii), April (sites iv) or May 2014 (sites v). The choice of March, April or May for snail collection was based on the fact that most infected snails, belonging to the overwintering generation, contained free cercariae within their body during this period. In 2015, samples performed in a total of 76 habitats were less than 50 snails because of the partial drying up of these sites during the sampling period and the burying of most overwintering adults into the soil. Owing to this difficulty in 2015, the total number of snails sampled during the two-year study was 17,647 (Table 2). In watered sites, snails were sampled using a 20-cm diameter sieve (mesh size, 0.2 cm). After collection, the snails were dissected under a stereomicroscope to detect the presence of trematode larval forms. Those of *C. daubneyi*, *F. hepatica* and *H. cylindracea* were directly identified because of our experience acquired with natural infections of *L. glabra* with these digenean species [25]. When larval forms of another trematode were detected, neutral red and/or Nile blue were used to study their morphology. Plagiorchiid cercariae were also heat killed and several dimensions were measured according to the reports by Combes [6] and Grabda-Kazubska [19]. Parasite identification was performed using taxonomic keys by several authors [13–15, 18, 26]. After identification, the prevalence of each parasite infection was determined.

**Table 2.** Number of *Lymnaea glabra* infected by each digenean species and prevalence of natural infection in five types of habitats located in the southern part of the Brenne Regional Natural Park, department of Indre, central France. *n*, total number of adult snails collected during the 2 years of study.

Parasite species	Number of infected snails (prevalence in %)					
	Drainage furrows (*n*: 3655)	Road ditches (*n*: 8491)	Pools (*n*: 2800)	Pond banks (*n*: 3045)	Brooks (*n*: 2256)	All sites (*n*: 17,647)
*Calicophoron daubneyi*	51 (1.3)	24 (0.2)	7 (0.2)	0	0	82 (0.4)
*Echinostoma revolutum*	0	0	0	2 (0.06)	0	2 (0.01)
*Fasciola hepatica*	71 (1.9)	43 (0.5)	2 (0.07)	1 (0.03)	0	117 (0.6)
*Haplometra cylindracea*	7 (0.1)	0	19 (0.6)	0	5 (0.2)	31 (0.1)
*Notocotylus* sp.	1 (0.02)	2 (0.02)	3 (0.1)	2 (0.06)	1 (0.04)	9 (0.05)
*Opisthoglyphe ranae*	0	0	11 (0.3)	32 (1.0)	0	43 (0.2)
*Plagiorchis* sp.	0	0	0	3 (0.09)	0	3 (0.01)
Immature infections						
with rediae	2 (0.04)	0	0	2 (0.06)	2 (0.08)	6 (0.03)
with sporocysts	9 (0.2)	2 (0.02)	7 (0.2)	5 (1.6)	2 (0.08)	25 (0.1)
Xiphidiocercariae (unidentified species)	0	0	0	2 (0.06)	1 (0.04)	3 (0.01)
Total number of infected snails (prevalence in %)	141 (3.8)	71 (0.08)	49 (1.7)	49 (1.6)	11 (0.4)	321 (1.8)

The first parameter was the number of parasite species in each type of snail habitat. The frequency of sites containing snails infected by a digenean was the second parameter and was determined for each type of habitat using the ratio: number of sites with infected snails/total number of snail habitats. Prevalence of each digenean infection and the overall prevalence of parasite species for each type of snail habitat were calculated using the ratio: number of infected snails/total number of sampled *L. glabra*. In the case of snail co-infections, prevalence was specified for each parasite species. A χ^2^ test was used to determine levels of statistical significance. Prevalences noted in the same type of habitats were first compared. In a second analysis, the differences between *C. daubneyi* values and those noted for *F. hepatica* in furrows, road ditches and pools were assessed because snail samples were collected over the same period of time (March). All the analyses were done using StatView 5.0 software (SAS Institute Inc., Cary, NC, USA). The nomenclature adopted by Correa et al. [7] and that by Jones [21] were used in the present study to identify lymnaeid and paramphistome species, respectively.

Of the 17,647 adult snails collected from the 206 habitats over the 2 years, three plagiorchiid, one echinostomatid, one fasciolid, one notocotylid and one paramphistomid species were found (Table 1). Immature infections without free cercariae were also noted. Those belonging to *C. daubneyi*, *F. hepatica* and *H. cylindracea* were easily recognized because of our experience and were classified with the corresponding snails harbouring mature infections. In contrast, those of the other digenean species were considered separately, taking into account the presence of rediae or secondary sporocysts. Xiphidiocercariae were also noted in the foot of several snails but their characteristics did not allow precise identification of the plagiorchiid species. Eleven double infections with *C. daubneyi* and *F. hepatica* were noted (data not shown).

Table 1 gives the distribution of habitats from which infected lymnaeids were collected. Snails harbouring larval forms of *F. hepatica* and/or *C. daubneyi* were found, respectively, in 14.5% and 11.6% of sites investigated. The percentages were lower for snails with *Opisthoglyphe ranae* Frölich, 1791 [16] (5.8%) and *H. cylindracea* (5.3%), and were less than 5% for individuals infected by a digenean of the other three species. Most snails infected with *F. hepatica* were collected from rainwater-draining furrows (45.9%), road ditches (12.7%) and pools (3.5%). Similar observations were noted for snails with *C. daubneyi* (37.8% of furrows, 8.1% of road ditches and 10.7% of pools). These pools were also the main habitats for snails infected with *H. cylindracea* (25.0%) and *O. ranae* (17.8%). The frequency of habitats with snails infected with other parasite species, particularly those with *Notocotylus sp*., was clearly lower. The highest number of parasite species was noted in pools located in meadows and on pond banks (five species in each case). During the 2 years of study, the presence of *F. hepatica* was regularly noted in snails living in 7 furrows (out of 17 habitats with infected *L glabra*) and 5 road ditches (out of 11). This was also observed for *C. daubneyi* (7 furrows/14, 3 road ditches/7), *H. cylindracea* (4 pools/7) and *O. ranae* (2 pools/5, 4 ponds/7). In the other types of habitats, the presence of snail infections was more sporadic (data not shown).

Table 2 gives values for each type of snail habitat and each digenean infection. Of the 17,647 *L. glabra* collected from the five types of habitats, a total of 321 infected snails were noted. The highest overall prevalence of all digenean infections was noted in furrows (3.8%), followed by pools (1.7%) and ponds (1.6%) by decreasing order. In the other two types of habitats, prevalence was < 1%. The percentage noted for each digenean infection varied with the type of habitat. In snails collected from furrows, prevalence of *F. hepatica* (1.9%) was slightly higher than that of *C. daubneyi* (1.3%) but this difference was not significant. Percentages greater than 0.5% were noted in pools for *H. cylindracea* and in ponds for *O. ranae*. In contrast, the prevalence for the other parasite species was less than 0.5%, whatever the type of habitat. If the values recorded for *F. hepatica* in the different types of habitats were compared, the prevalence of this digenean infection was significantly greater (χ^2^ = 105.16, *p* < 0.001) in snails living in furrows than in road ditches and pools. In the same way, the value noted for *C. daubneyi* infection was significantly higher (χ^2^ = 93.28, *p* < 0.001) in snails living in furrows than in road ditches and pools.

Owing to the limited geographic range of *L. glabra* in Western Europe, from Scandinavia up to Spain [20] and little information available on parasitological fauna in this snail, the results noted in the present study were compared with reports by Rondelaud [27] on *L. glabra* from the Limousin region and Rondelaud et al. [28] on *Galba truncatula* O.F. Müller, 1774 [25] from the Brenne Regional Natural Park. Compared to *L. glabra* samples collected by Rondelaud [27], the number of parasite species was greater in the present study (7 species instead of 5) but was lower than that (10 species) noted in *G. truncatula* originating from the same six French municipalities [28]. The presence of numerous pools and ponds in the Brenne Regional Natural Park might explain this increase in the number of parasite species in our *L. glabra*. In contrast, the difference noted between *G. truncatula* and *L. glabra* from the same six municipalities is more difficult to interpret. In our opinion, this discrepancy might be mainly due to the situation of snail habitats along the same hydrographical network: those colonized by *G. truncatula* were often located at the peripheral extremity of rainwater-draining and open drainage furrows, for example, while those inhabited by *L. glabra* in the same furrows were found more downstream [11]. However, another explanation based on the abundance of both lymnaeid species in the Brenne Regional Natural Park (the size of *L. glabra* populations was clearly greater than that of *G. truncatula* populations [30]) cannot be completely excluded.

Most *L. glabra* infected with *F. hepatica* and/or *C. daubneyi* were found in snail samples collected from furrows and this result was consistent with the report by Rondelaud et al. [28] on *G. truncatula*. In contrast, the *L. glabra* infected by either digenean were more numerous in road ditches and scarcer in pools. In contrast, the distribution of infected *G. truncatula* in both these types of habitats was the opposite [28]. Three points may explain this difference, (i) the presence of more *L. glabra* populations in road ditches which bordered cattle- or sheep-grazed meadows than in pools [30], (ii) the flow of runoff coming from these meadows into these ditches, particularly in winter and spring, and (iii) the location of *L. glabra* among amphibious and/or floating vegetation in the center of pools, while the *G. truncatula* population was often found on the margins of these sites (unpublished data). The presence of the echinostomatid and plagiorchiid species in pools and ponds was consistent with the report of Rondelaud et al. [28] on the *G. truncatula* from the same French municipalities. Similarly, the findings of *Notocotylus sp.* in snails collected from the five types of habitats confirm presence of this parasite in local *G. truncatula* [28].

In *L. glabra*, the prevalence of *F. hepatica* infection varied with the type of habitat and was significantly higher in furrow-collected snails than in those coming from road ditches and pools. A similar finding was also noted for prevalence of *C. daubneyi*. These variations in both prevalences may be explained by two points: (i) both digeneans often used the same definitive and intermediate hosts for their life cycle [22], and (ii) the co-infection of pre-adult *L. glabra* (4 mm in shell height) with both types of miracidia often resulted in complete larval development of *F. hepatica*, *C. daubneyi* or both [1–4]. Prevalence of *H. cylindracea* (0.6%) in pool-collected *L. glabra* was clearly higher than that (0.1%) reported by Rondelaud [27] for the same lymnaeid in wild watercress beds. This difference might be due to the fact that infected frogs (the definitive host of this digenean) would infect numerous snails during their stay in pools. The finding of numerous *H. cylindracea*-infected *G. truncatula* in the populations studied by Vignoles et al. [29] between 2001 and 2009 on acid soils supports this last hypothesis. In the present study, larval forms of four other digenean species were also found in the body of *L. glabra* (Table 2). The most frequent was *O. ranae* with 0.3% and 1% prevalences in pools and ponds, respectively. As no larval forms of these four digeneans were reported by Rondelaud [27] in the *L. glabra* from the Limousin region, this paper was the first report of this lymnaeid as intermediate host for *Echinostoma revolutum* Frölich, 1802 [17], *Notocotylus sp.*, *O. ranae* and *Plagiorchis sp.*

In conclusion, the *L. glabra* living in the Brenne Regional Natural Park acted as intermediate hosts in the life cycles of seven digeneans. The highest prevalences were noted for *C. daubneyi* and *F. hepatica*. The distribution and prevalence of each parasite species were dependent on the type and, consequently, the location of each snail habitat.
